# Receptive Arts Engagement Activities and Depressive Symptoms Among Older Adults: Serial Indirect Associations Through Leisure-Related Relaxation and Subjective Well-Being

**DOI:** 10.3390/bs16091572

**Published:** 2026-09-04

**Authors:** Xu Song, Jihoon Jang

**Affiliations:** Global Fine Arts Department, Kyonggi University, Suwon 16227, Republic of Korea; songxukgu@outlook.com

**Keywords:** art engagement activities, depression symptoms, older adults, subjective well-being, serial mediation

## Abstract

Depressive symptoms are a prevalent mental health concern among older adults. This study examined whether the frequency of receptive arts engagement was associated with depressive symptom scores through leisure-related relaxation and subjective well-being in a specified serial mediation model. Using cross-sectional data from the 2023 Chinese General Social Survey (CGSS), we analyzed 2332 adults aged 60 years or older. Hierarchical regression and bias-corrected bootstrap mediation analyses with 5000 resamples were conducted to examine the proposed model. We found that more frequent art activities were significantly associated with fewer depressive symptoms. Three indirect associations were examined. The indirect association through leisure and relaxation alone was not significant, whereas the association through subjective well-being was the largest of the three tested pathways. A small serial indirect association through leisure and relaxation and subjective well-being was also identified. This model-specific pathway should not be interpreted as causal or as the primary mechanism of arts engagement. These findings describe one possible pathway linking receptive arts engagement to depressive symptoms among older adults. Further research should examine this pathway alongside other psychological, biological, social, and behavioral processes.

## 1. Introduction

The world is experiencing a rapid and unprecedented wave of population aging as many people grow older. This important shift in demographics has implications for socio-economics, public health, and family care (Dann, 2014; Longevity, 2021). Elderly mental health issues are one of the most important health problems of aging. Among these, depression, as one of the most prevalent mental disorders, significantly compromises older adults’ quality of life and subjective well-being (Blazer, 2003; Cai et al., 2022). In older adults, depression may manifest as persistent low mood, loss of interest, and anhedonia. Cognitive impairment is also part of it and often causes discomfort (physical) and impairment in social participation. Consequently, it increases the chances of chronic diseases and disabilities. Most importantly, it increases the chance of death (Wilkins, 2008; Szymkowicz et al., 2023). Older adults warrant specific attention because mental health, subjective well-being, and social connection are closely intertwined in later life. Retirement, bereavement, children leaving home, and shrinking social networks can reduce meaningful social roles and increase isolation and loneliness (Fan, 2016; Simard & Volicer, 2020; Zubatsky et al., 2020). In this context, depression can further compromise cognitive, physical, and social functioning, independence, and quality of life (Wilkins, 2008; Szymkowicz et al., 2023). Accessible non-pharmacological activities that can be incorporated into everyday life are therefore particularly relevant for this population. These age-specific vulnerabilities and practical needs motivate our focus on depressive symptoms and subjective well-being among older adults. Social connection provides important population-level context, although it was not measured as a mediator in the present model. Clinical major depressive disorder should be distinguished from the single mood-frequency indicator analyzed in this study. Under DSM-5-TR criteria, a major depressive episode requires a cluster of symptoms to be present during the same period of at least two weeks and to cause clinically significant distress or impairment in social, occupational, or other important areas of functioning (American Psychiatric Association, 2022).

Arts-related activities encompass at least three distinct contexts: everyday receptive engagement (e.g., listening to music or viewing artworks), active participation (e.g., painting, singing, dance, or drama), and structured arts-based programs or art therapy delivered with therapeutic goals. These forms differ in participant role, setting, social context, intensity, and intended purpose, so findings from one context cannot be assumed to apply to another. Prior studies have associated both everyday cultural engagement and structured arts programs with mental-health and well-being outcomes in older adults (Cohen et al., 2007; Fields et al., 2019; McQuade & O’Sullivan, 2023). In the present study, however, the CGSS exposure is limited to self-reported receptive engagement during leisure time. Evidence on active programs and art therapy is used only to provide broader context and is not treated as directly equivalent to the measured exposure.

Leisure activities are generally thought to facilitate relaxing and pleasurable experiences. Whether it is wandering through nature (Zurawik, 2020) or spending time in community green spaces (Mansor et al., 2012), such activities contribute to enhancing our sense of relaxing and pleasurable experiences (Dashper & King, 2021). According to Liu and Yu (2014), relaxation and satisfaction from leisure may be an important way to improve subjective well-being. Leisure experiences that generate satisfaction are positively correlated with subjective well-being and life satisfaction, whether the activity is music (Laukka, 2006), cooking (Güler & Haseki, 2021), or any social leisure activity (Nielsen et al., 2021). Cotter and Pawelski (2021) reviewed evidence indicating that art museum visitation and museum program participation are associated with flourishing-related outcomes, including reduced ill-being and enhanced well-being. The mechanisms underlying these benefits are multifaceted and extend beyond leisure-related relaxation. Arts engagement may influence mental health through interacting psychological, neurophysiological, social, and behavioral pathways. These pathways can involve emotion regulation and reward processing; stress-related autonomic, neuroendocrine, and immune responses; and social connection (Fancourt et al., 2021; Fancourt et al., 2026). Within this broader framework, leisure and relaxation represent one possible psychological pathway rather than a comprehensive explanation.

Other researchers have generally found that engaging in art activity positively impacts the mental well-being of older adults. What remains unclear, however, is the ‘how’ behind such positive outcomes. Most studies of art activities and depression have focused on outcomes rather than on the mechanisms underlying these effects. First, art activities are often categorized as recreation. The term “relaxation” within the context of psychological experiences that impact depression through art activities is less precise and not explicitly tested in the artistic context. Although relaxation, as a key recovery tool, has received considerable attention in work stress research (Xanthopoulou et al., 2017; Barber et al., 2019), empirical work is needed that tests relaxation as a mediating variable in older adults’ day-to-day leisure activities. Secondly, although subjective well-being is often regarded as a positive outcome of art activities, its function as a key bridge linking leisure experience and depression improvement, and its place on the mediating chain, requires greater precision. The DRAMMA model identifies six psychological experiences through which leisure participation may support well-being: detachment, relaxation, autonomy, mastery, meaning, and affiliation (Kujanpää et al., 2020). In this study, the model provides a conceptual basis for considering relaxation as one potential leisure-related experience associated with subjective well-being. Accordingly, we examine the statistical associations among the frequency of participation in art activities, leisure-related relaxation, subjective well-being, and depressive symptoms, including the hypothesized indirect associations among these variables. Because the data are cross-sectional, these associations should not be interpreted as evidence of temporal ordering or causality. Importantly, the CGSS item used below does not directly measure the DRAMMA relaxation experience as a subjective state. It records how frequently respondents rested and relaxed during free time. DRAMMA is therefore used as a heuristic framework for identifying a potentially relevant recovery-related domain, rather than as a construct-validating measurement model tested by the present data.

This study focuses on four domains that can be operationalized in the CGSS 2023 and that correspond to distinct roles in the proposed model: receptive arts engagement as the exposure, the reported frequency of resting and relaxing during free time as a behavioral indicator related to—but not equivalent to—the DRAMMA relaxation experience, subjective well-being as a positive evaluative dimension of mental health, and depressive symptom frequency as the outcome. The DRAMMA framework motivates examination of whether a relaxation-related leisure indicator is associated with subjective well-being, but the single CGSS item does not provide a direct test of psychological recovery. We therefore test whether more frequent receptive arts engagement is associated with lower depressive symptom scores directly and indirectly through (1) the frequency of resting and relaxing during free time, (2) subjective well-being, and (3) their serial statistical association. Cross-sectional CGSS 2023 data are used to estimate these associations with hierarchical regression and bootstrap confidence intervals. The study’s primary focus is the magnitude and uncertainty of these specified associations, not the efficacy of art therapy or the causal identification of psychological, biological, social, or behavioral mechanisms.

## 2. Literature Review

### 2.1. Receptive Arts Engagement and Depressive Symptoms Among Older Adults

In the present study, the term art activities refer specifically to everyday receptive arts engagement, operationalized as listening to music or appreciating artworks during free time. This differs from art therapy, which comprises structured arts-based interventions with explicit therapeutic aims. Observational evidence on receptive cultural engagement is therefore more directly comparable to our exposure than clinical arts-based interventions. Fancourt and Tymoszuk (2018) reported that visits to museums, galleries, cinemas, theatres, concerts, and operas were associated with a lower risk of incident depression in adults aged 50 years and older. Systematic reviews of group arts interventions and art therapy have also reported reductions in depressive symptoms among older adults (Jenabi et al., 2022; Quinn et al., 2025). However, we treat this intervention evidence as complementary rather than directly equivalent because these interventions differ from the receptive, non-clinical activities measured in the CGSS. Based on this evidence, we propose:

**H1.**  *More frequent receptive arts engagement is associated with lower depressive symptom scores among older adults.*

### 2.2. Subjective Well-Being as a Proposed Statistical Mediator

The broader literature on arts engagement and well-being includes heterogeneous modalities, ranging from receptive cultural participation to active and therapeutic programs. Studies of singing, museum visitation, group drumming, dance, photography, drama, and painting have reported associations with subjective well-being and depressive or anxiety symptoms (Mak et al., 2024), while other reviews encompass visual arts, drama, literature, and music (Fioranelli et al., 2023). These activities differ in format, intensity, and therapeutic purpose and are not treated as equivalent exposures in the present study. The narrower rationale for our model is that subjective well-being has been associated with both cultural engagement and depressive symptoms in prior research (M. Zhang et al., 2025). Together, this evidence provides a rationale for examining subjective well-being as a statistical mediator. It does not establish that receptive arts engagement increases well-being or that higher well-being causally reduces depressive symptoms in the present cross-sectional data. We therefore propose:

**H2.**  *More frequent receptive arts engagement is indirectly associated with lower depressive symptom scores through higher subjective well-being.*

### 2.3. Leisure-Related Relaxation as a Proposed Statistical Mediator

Beyond subjective well-being, a relaxation-related leisure indicator may represent another statistical pathway linking receptive arts engagement with depressive symptoms. The DRAMMA model identifies relaxation as one of several psychological experiences through which leisure participation may support well-being, while broader frameworks emphasize that leisure activities may operate through interacting psychological, biological, social, and behavioral processes (Fancourt et al., 2021). Consistent with this interpretation, a systematic review reported reductions in stress-related outcomes in 30 of 37 studies of creative arts interventions (Martin et al., 2018). The present CGSS item, however, measures the frequency of resting and relaxing rather than the subjective DRAMMA relaxation experience. Moreover, receptive arts engagement is itself a form of leisure activity, whereas the proposed mediator is a separate item concerning resting and relaxing during free time. The modeled path should therefore not be interpreted as a transition from a non-leisure behavior to a distinct leisure behavior, or as evidence that arts engagement temporally causes relaxation. Instead, it tests whether respondents who report more frequent receptive arts engagement also report more frequent resting and relaxing, and whether this pattern is statistically associated with depressive symptoms. Evidence that this behavioral frequency indicator independently mediates the association between arts engagement and depressive symptoms remains limited; other research suggests that leisure–mental-health associations may involve factors such as social support and perceived stress (C. Zhang et al., 2021). We therefore propose:

**H3.**  *More frequent receptive arts engagement is indirectly associated with lower depressive symptom scores through a higher reported frequency of resting and relaxing during free time. This hypothesis concerns a statistical indirect association and does not assume that the CGSS item directly measures the DRAMMA psychological relaxation experience.*

### 2.4. Proposed Serial Indirect Association Through Relaxation and Subjective Well-Being

Building on the preceding literature, we examine one specific indirect association among receptive arts engagement, leisure-related relaxation, subjective well-being, and depressive symptoms. The proposed sequence does not assume that relaxation is sufficient, primary, or universal. Instead, relaxation is treated as one possible leisure-related experience within a broader set of psychological, biological, social, and behavioral processes that may connect arts engagement with mental health. In DRAMMA terms, relaxation is one of several experiences through which leisure may be associated with well-being. In the present model, however, the first proposed mediator is only a behavioral frequency indicator related to that domain; therefore, the serial path is a theory-informed statistical approximation rather than a direct test of a DRAMMA recovery process.

Evidence on leisure and depression points to multiple possible mediators rather than a single relaxation-based mechanism. Using cross-sectional data from 10,058 Chinese adults aged 65 years and older, Cui et al. (2025) examined dietary measures and cognitive function as mediators between leisure activity and depression. Their models included separate and sequential indirect associations through dietary diversity, a plant-based diet index, and cognitive function. These findings illustrate that leisure-depression associations may involve distinct behavioral and cognitive processes. However, that study did not test the relaxation-to-subjective-well-being pathway proposed here. Qualitative arts research has described relaxation, stress relief, and emotional regulation as possible experiences during artistic activity (Dunphy et al., 2019). Such evidence provides contextual support but does not establish a neurophysiological sequence or identify relaxation as the primary mechanism. Accordingly, our hypothesis is limited to one statistical indirect association.

**H4.**  *More frequent receptive arts engagement is indirectly associated with fewer depressive symptoms through a higher reported frequency of resting and relaxing during free time and higher subjective well-being. This hypothesis concerns one theory-informed statistical pathway; it neither treats the CGSS resting/relaxing item as a direct measure of the DRAMMA relaxation experience nor identifies relaxation as the primary or universal mechanism of arts engagement or art therapy.*

**Figure 1:**
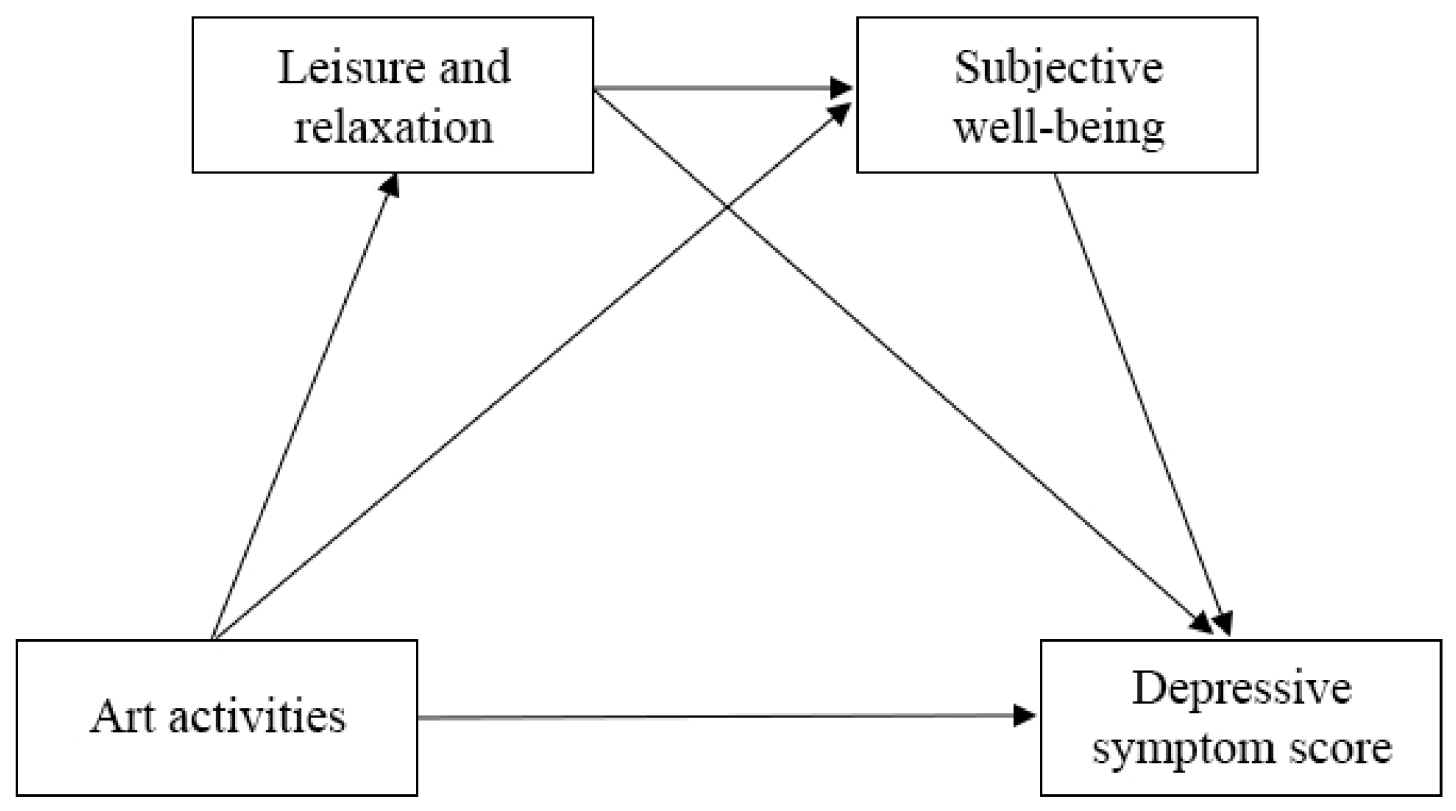
Proposed chain-mediation model linking art activities to the depressive symptom score. All paths terminating at the depressive symptom score represent hypothesized inverse associations; the arrows identify the modeled outcome and do not imply an increase in depressive symptoms.

In summary, the study tests whether leisure-related relaxation and subjective well-being form a serial statistical indirect association between receptive arts engagement and depressive symptoms (Figure 1).

## 3. Methods

### 3.1. Research Design and Data Source

This study used the publicly released 2023 wave of the Chinese General Social Survey (CGSS 2023) (Chinese General Social Survey, n.d.). The CGSS is a repeated cross-sectional survey administered by the National Survey Research Center (NSRC) at Renmin University of China. Established in 2003, it monitors changes in social structure and quality of life in urban and rural China. It also provides standardized data for academic research, policy analysis, and international comparisons. This survey data systematically collects information in the social, economic, cultural and health dimensions for the population in China (Bian & Li, 2012). The target population comprises civilian adults aged 18 years or older in mainland China. The CGSS uses a multistage, stratified probability-proportional-to-size sampling design. In 2023, the survey obtained 11,326 valid responses through two data-collection modes. The 2023 dataset contains 439 variables covering sociodemographic characteristics, housing, health, migration, lifestyle, social attitudes, class identity, labor markets, social security, and family life. The anonymized dataset was publicly released by NSRC and obtained through the Chinese National Survey Data Archive (National Survey Research Center, 2025). Because this study used de-identified public data, no additional ethical approval was required.

### 3.2. Study Sample

**Figure 2:**
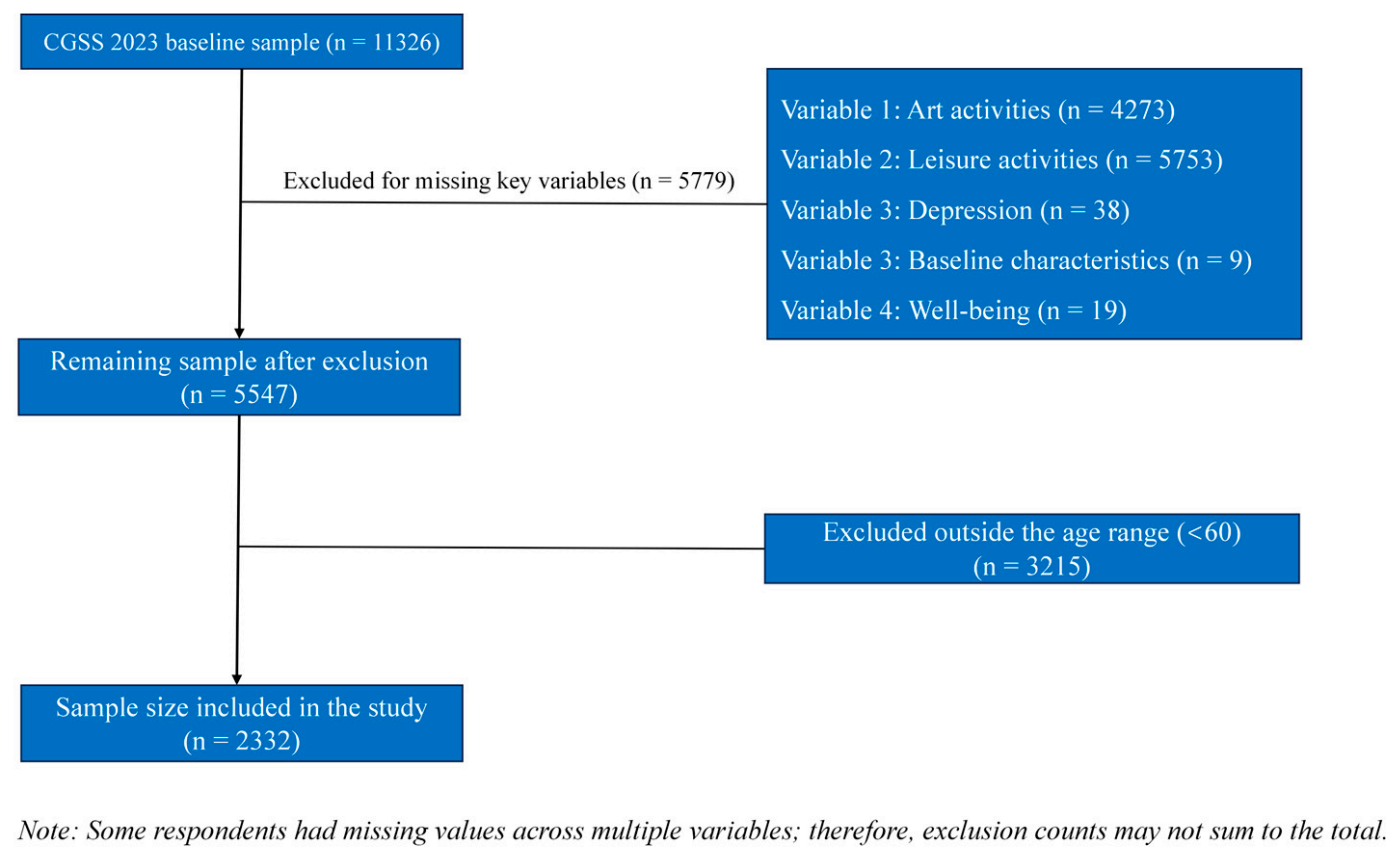
Sample screening process.

The CGSS 2023 public-use dataset contained 11,326 respondents. Of these, 5779 lacked at least one variable required for the present analysis. Specifically, 4273 had no valid receptive arts-engagement response, 5753 had no valid leisure-related relaxation response, 38 had no valid depressive-symptom response, 19 had no valid subjective well-being response, and 9 lacked at least one baseline covariate. These counts overlap because some respondents had missing values for multiple variables. The unusually large number of unavailable leisure-related relaxation responses may reflect questionnaire routing or structural non-administration rather than ordinary item nonresponse. Complete-case restriction left 5547 respondents. After excluding 3215 respondents younger than 60 years, the final analytical sample comprised 2332 older adults (Figure 2). Although the original CGSS used a national probability sampling design, the final complete-case analytical sample cannot automatically be considered nationally representative.

### 3.3. Measures

The measures were limited to the four domains specified in the conceptual model. The arts item served as the exposure, leisure-related relaxation and subjective well-being as candidate mediators, and depressive symptom frequency as the outcome. These roles were assigned from the theoretical rationale in the Introduction rather than from the observed correlations. The CGSS did not provide equivalent measures of the broader biological, social, or behavioral processes discussed in the literature; those processes were therefore not included in the statistical model.

#### 3.3.1. Art Activities

The exposure, termed receptive arts engagement frequency, was operationalized using the CGSS 2023 leisure item asking how often respondents listened to music or appreciated artworks during their free time in the past year. The original response categories were 1 = every day, 2 = several times a week, 3 = several times a month, 4 = several times a year or less, and 5 = never. Responses were reverse-coded so that higher scores indicated more frequent engagement (1 = never to 5 = every day). In this observational study, exposure or “dose” therefore refers only to self-reported frequency. The CGSS item did not record duration per occasion, intensity, cumulative hours, specific content or setting, facilitator involvement, or therapeutic intent; consequently, it cannot be used to estimate a clinical treatment dose or a dose–response relationship. The measure captures receptive, non-clinical leisure engagement and does not assess active art-making, participation in organized arts programs, or art therapy.

#### 3.3.2. Leisure and Relaxation

The CGSS 2023 item asked respondents how frequently they rested and relaxed during their free time in the past year. Responses were recorded on a 5-point scale (1 = “Never”, 5 = “Very Frequent”), with higher scores indicating more frequent resting and relaxing. This item measures the reported frequency of a leisure-time behavior; it does not assess the subjective quality, depth, or effectiveness of relaxation and is not a validated measure of the psychological relaxation experience specified in the DRAMMA model. We therefore refer to it as a leisure-related resting/relaxing frequency indicator and interpret it as an imperfect behavioral proxy for the broader relaxation domain.

#### 3.3.3. Subjective Well-Being

Subjective well-being (SWB) was assessed using the standardized subjective well-being measurement item from the CGSS 2023 questionnaire: “Overall, do you feel happy with your current life?” A 5-point Likert scale was used (1 = “very unhappy”, 5 = “very happy”), with higher scores indicating stronger subjective well-being. This single-item measure captures respondents’ overall evaluative happiness but does not represent the broader multidimensional construct of subjective well-being, which typically includes both affective and cognitive components. Therefore, it should be interpreted as a global indicator of perceived happiness rather than a comprehensive assessment of subjective well-being. This single-item indicator is widely used in studies of subjective well-being among older adults based on large-scale survey data, and its face validity and convergent validity have been well-validated (Ji et al., 2025).

#### 3.3.4. Depressive Symptoms

In the CGSS 2023 questionnaire, we assessed depressive symptoms by using the mood state measurement items. The outcome variable reflects the self-reported frequency of depressed or frustrated mood rather than clinically diagnosed depression. The variable in this study was back-coded. The occurrence of depressive symptoms was measured in the subjects using the 5-point Likert scale (1 = “never”, 5 = “always”) in which higher scores indicate more frequent occurrence of depressive symptom. The item did not assess whether symptoms were present continuously for at least two weeks, the broader symptom cluster required for a major depressive episode, or clinically significant distress or impairment in occupational functioning, social relationships, or other important areas of life as specified in DSM-5-TR (American Psychiatric Association, 2022). Although such single-item mood indicators are commonly used in large population surveys because of their feasibility, they should be interpreted as indicators of self-reported mood frequency rather than clinical depression. In large social surveys, using single-item frequencies to assess depressive status is widely used to conduct research on mental health in elderly (Leng et al., 2024).

### 3.4. Statistical Analysis

The analysis followed the four-domain model specified above. First, descriptive statistics were calculated for each study variable, and Pearson correlation coefficients were used to describe unadjusted bivariate associations. These correlations were treated as preliminary descriptive evidence; they were not interpreted as tests of temporal ordering or causality and were not used to add or remove domains from the conceptual model.

The second step involved estimating a series of regression equations corresponding to the hypothesized mediation model rather than conducting a conventional hierarchical blockwise regression analysis. Four regression equations were estimated to examine the associations among receptive arts engagement, leisure-related relaxation, subjective well-being, and depressive symptoms.

In all equations, gender (0 = male, 1 = female), age (years), education level, marital status, household registration location, and self-rated health were entered as covariates. Education, household registration location, and self-rated health were treated as ordinal variables with higher scores indicating higher educational attainment, greater residential mobility, and better perceived health, respectively. Marital status was represented by a set of dummy variables, with married (first marriage) serving as the reference category. These covariates were included in every regression equation of the mediation model. The covariate set was specified a priori to address several broad sources of confounding that could be associated with both receptive arts engagement and depressive symptoms. Age and gender represent demographic and life-course differences; education and household registration location capture aspects of socioeconomic position and differential access to resources; marital status reflects household and social context; and self-rated health captures general health-related selection into arts engagement. This parsimonious adjustment set was limited to covariates available for the complete-case analytical sample and should not be regarded as exhaustive control of confounding.

The mediation model was estimated in Mplus 8.0 using the robust maximum likelihood estimator (MLR), and all reported regression coefficients are standardized estimates (StdYX). The five-point study variables were treated as approximately continuous because they contained five ordered response categories and were analyzed using MLR estimation, an approach widely adopted in structural equation modeling. Bias-corrected bootstrap confidence intervals based on 5000 resamples were used to evaluate indirect effects. An indirect effect was considered statistically significant when the corresponding 95% confidence interval did not include zero.

Because the CGSS 2023 employed a multistage stratified probability sampling design, the analyses accounted for the complex survey design in Mplus 8.0. Sampling weights were incorporated using the WEIGHT option to obtain nationally representative parameter estimates. Primary sampling units were specified using the CLUSTER option, and sampling strata were specified using the STRATIFICATION option. All mediation models were estimated using TYPE = COMPLEX, which applies a sandwich (Huber–White) estimator to adjust standard errors and chi-square statistics for the complex sampling design. Bootstrap confidence intervals (5000 resamples) were then used to evaluate the statistical significance of the indirect effects. Parameter estimates were obtained using robust maximum likelihood estimation (MLR), providing standard errors that are robust to non-normality and complex sampling. The third step used a bias-corrected nonparametric bootstrap method to estimate the indirect associations (Hayes, 2017; Preacher & Hayes, 2008). Mplus 8.0 was used with art activities as the independent variable, the depressive symptom score as the dependent variable, and relaxation and subjective well-being as the first and second mediating variables, respectively. A total of 5000 bootstrap resamples were used to construct 95% confidence intervals (CIs). An indirect estimate was considered statistically significant when its bootstrap 95% CI did not include 0 (Preacher & Hayes, 2008). The three indirect paths were path 1 (art activities → leisure and relaxation → depressive symptom score), path 2 (art activities → subjective well-being → depressive symptom score), and path 3 (art activities → leisure and relaxation → subjective well-being → depressive symptom score). Negative indirect estimates for paths terminating at this outcome indicate associations with fewer depressive symptoms. The arrows specify the statistical model and do not imply an increase in symptoms or establish causal effects.

The primary analyses were conducted using complete cases because estimation of the specified indirect associations required joint observations on all focal variables and covariates. Conventional multiple imputation was not applied because the large block of unavailable leisure-related relaxation responses may represent structural non-administration. Imputing responses for participants who were not administered the item would require unverifiable assumptions about questionnaire routing and module assignment. The results should therefore be interpreted as applying to respondents with complete observations on all study variables. Comparisons between included and excluded respondents and design-based sensitivity analyses should be undertaken once the relevant questionnaire-routing information is available.

## 4. Results

### 4.1. Sample Characteristics

Regarding the basic characteristics of the sample (as shown in Table 1), there were slightly more women than men (52.80% women and 47.20% men), and the average age was 69.91 years (SD = 6.84). Most marital statuses were first marriages with a spouse (67.80%), and many registered residents were from the same township (79.40%). Regarding educational attainment, the percentages of those with primary school (29.90%) and junior high school (26.70%) education levels are relatively low, which is basically consistent with the reality of China’s elderly population. Regarding physical health, the highest percentage (31.90%) rated themselves as “average” and the second highest as “relatively healthy” (24.70%).

### 4.2. Descriptive Statistics and Correlation Analysis

Table 2 presents the Pearson correlation coefficients and descriptive statistics for each study variable. The mean scores were 2.16 (SD = 1.04) for art activities, 3.76 (SD = 0.98) for leisure and relaxation, 3.91 (SD = 0.89) for subjective well-being, and 2.25 (SD = 1.20) for depressive symptoms. Higher depressive symptom scores indicate more frequently reported symptoms.

Art activities were negatively correlated with depressive symptom scores (r = −0.136, *p* < 0.001) and positively correlated with subjective well-being (r = 0.175, *p* < 0.001) and leisure and relaxation (r = 0.110, *p* < 0.001). Leisure and relaxation were positively correlated with subjective well-being (r = 0.096, *p* < 0.001), but its correlation with depressive symptom scores was not significant (r = −0.017, *p* > 0.05). Subjective well-being was negatively correlated with depressive symptom scores (r = −0.302, *p* < 0.001). Thus, the negative correlations with the outcome indicate associations with fewer depressive symptoms rather than an increase in symptoms. These patterns provided a preliminary basis for examining the proposed indirect associations.

### 4.3. Hierarchical Regression Analysis

This research developed four hierarchical regression models (refer to Table 3) to examine the statistical associations between receptive arts engagement and depressive symptoms among older adults and the specified indirect pathways. All models controlled for gender, age, education level, marital status, place of household registration, and physical health status.

Model 1 used the depressive symptom score as the dependent variable. More frequent art activities were associated with lower depressive symptom scores (β = −0.042, SE = 0.019, *p* = 0.025). Thus, the negative coefficient corresponds to fewer reported symptoms. In Model 2, art activities were positively associated with leisure and relaxation (β = 0.101, SE = 0.021, *p* < 0.001). In Model 3, art activities and leisure and relaxation were positively associated with subjective well-being (art activities: β = 0.138, SE = 0.021, *p* < 0.001; leisure and relaxation: β = 0.084, SE = 0.020, *p* < 0.001). In Model 4, the direct association between art activities and depressive symptoms was not significant after leisure and relaxation and subjective well-being were included (β = −0.013, SE = 0.019, *p* = 0.472). Subjective well-being remained negatively associated with depressive symptoms (β = −0.193, SE = 0.019, *p* < 0.001), whereas leisure and relaxation was not significantly associated with the outcome (β = −0.002, SE = 0.018, *p* = 0.907).

### 4.4. Chain Mediation Analysis

**Figure 3:**
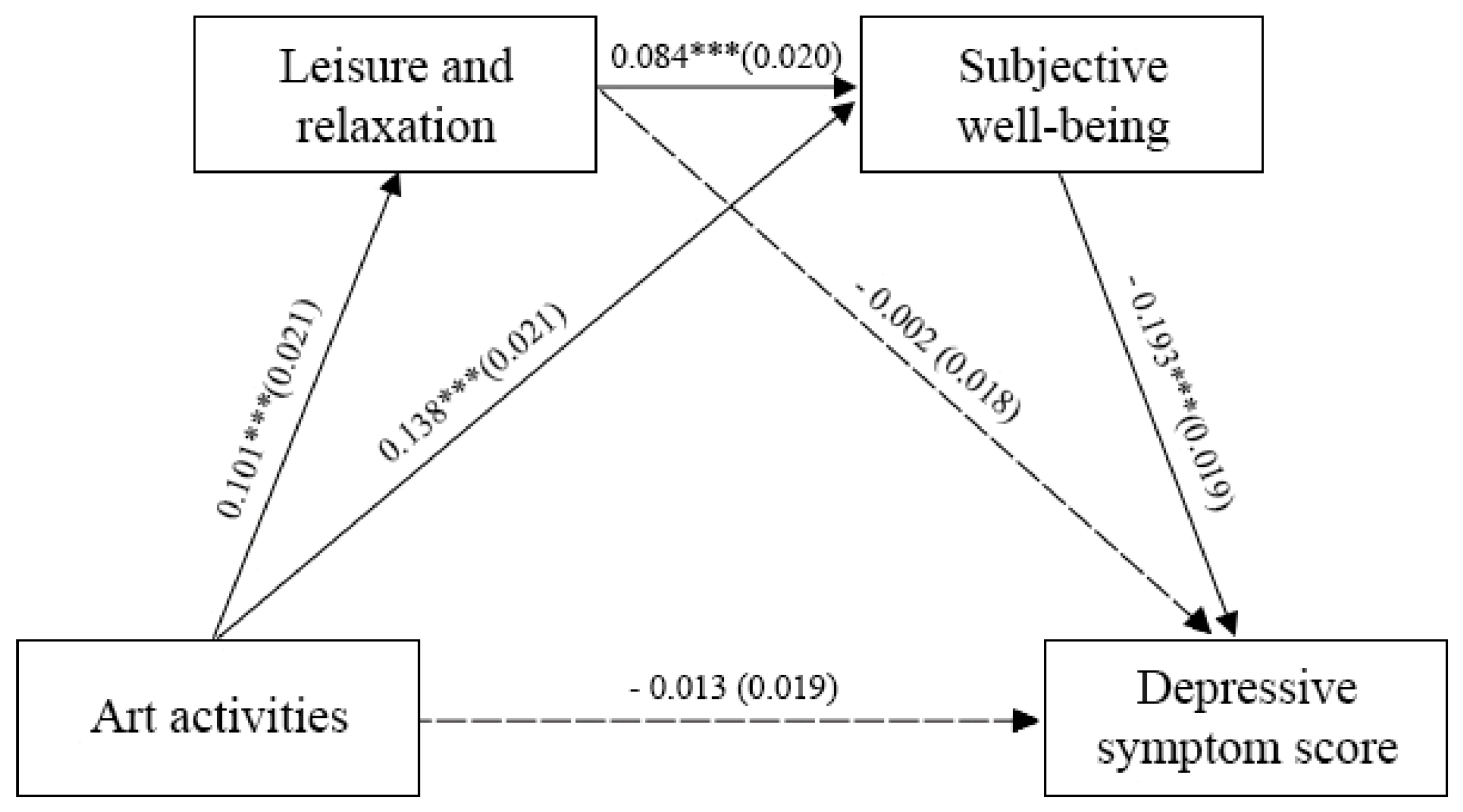
Estimated chain-mediation model. Note. Values are standardized regression coefficients, with standard errors in parentheses. Solid arrows indicate statistically significant paths, whereas dashed arrows indicate nonsignificant paths. *** *p* < 0.001. The outcome box represents the depressive symptom score; negative coefficients on paths terminating at this outcome indicate associations with lower depressive symptom scores.

The bootstrap analysis estimated the direct and indirect associations in the proposed chain-mediation model. Figure 3 and Table 4 show their directions and magnitudes. Negative coefficients on paths terminating at the depressive symptom score indicate associations with fewer depressive symptoms.

The overall association between art activities and depressive symptoms was negative (effect = −0.042, SE = 0.019, 95% CI [−0.078, −0.005]), indicating that more frequent art activities were associated with lower depressive symptoms scores. The total indirect association was −0.028 (SE = 0.005, 95% CI [−0.040, −0.019]), and its confidence interval did not include 0.

The three indirect associations were as follows. Indirect pathway 1 (art activities → leisure and relaxation → depression) was not significant (effect = 0.000, SE = 0.002, 95% CI [−0.004, 0.004]). Indirect pathway 2 (art activities → subjective well-being → depression) had the largest indirect effect among the three tested pathways (effect = −0.027, SE = 0.005, 95% CI [−0.037, −0.018]). Indirect pathway 3 (art activities → leisure-related relaxation → subjective well-being → depressive symptoms) was statistically significant but very small in magnitude (standardized indirect effect = −0.002, SE = 0.001, 95% CI [−0.003, −0.001]). Although the confidence interval excluded zero, the estimated effect size indicates that this pathway explains only a limited proportion of the overall association between receptive arts engagement and depressive symptoms. Therefore, its statistical significance should not be interpreted as evidence of substantial practical impact.

The direct association between receptive arts engagement and depressive symptoms was not statistically significant after the two proposed mediators were included (effect = −0.013, SE = 0.019, 95% CI [−0.050, 0.023]). This nonsignificant coefficient does not demonstrate that the direct effect is zero. Among the tested indirect associations, the pathway through subjective well-being alone was the largest, whereas the serial pathway involving leisure-related relaxation and subjective well-being was statistically significant but very small. Thus, H2 and H4 received statistical support, whereas H3 was not supported. Within the specified statistical model, most of the observed association was linked to the pathway involving subjective well-being. These estimates describe model-specific associations and do not establish temporal or causal mediation.

## 5. Discussion

This study used CGSS 2023 data to examine associations among four measured domains in older adults: receptive arts engagement, leisure-related relaxation, subjective well-being, and depressive symptoms. More frequent engagement was associated with fewer depressive symptoms. Among the three indirect associations specified in advance by the conceptual model, the pathway through subjective well-being alone was the largest, the serial pathway through relaxation and subjective well-being was small, and the pathway through relaxation alone was not significant. The analysis therefore supports a bounded conclusion about associations among these measured variables; it does not identify a comprehensive mechanism through which arts engagement affects mental health.

The inverse association between receptive arts engagement and depressive symptom scores is consistent with prior observational evidence on cultural engagement (Fancourt & Tymoszuk, 2018; McQuade & O’Sullivan, 2023). However, the broader literature also includes active arts programs, group interventions, and art therapy, which are not directly equivalent to the CGSS exposure. Because the present measure captured only listening to music or appreciating artworks during free time, the findings should not be treated as evidence of the efficacy of active arts programs or art therapy. Their contribution is limited to population-level correlational evidence concerning everyday receptive arts engagement.

Among the three tested indirect associations, the pathway through subjective well-being alone had the largest estimated magnitude. This result supports the inclusion of subjective well-being as a central domain in the specified model, but it does not establish that arts engagement causally increases well-being or that well-being protects against depression. Because the focal variables were measured at the same time using self-reports, reverse directionality, shared method variance, and unmeasured confounding remain plausible. This means that self-actualization, social support, and other proposed processes were not measured and should not be inferred from the observed indirect association.

Furthermore, this study reveals the mediating role of subjective well-being in the relationship between art engagement and depression. Our model shows that art engagement has no significant direct effect on depression; its protective effect is achieved by enhancing subjective well-being. This finding shifts the research perspective from the traditional “problem-solving” model (i.e., directly reducing depression) to the “strengths-building” model (i.e., actively enhancing subjective well-being). Art activities are not simply a form of entertainment to distract from negative emotions, but rather an activity that can inspire positive feelings, create meaning, and promote self-actualization, thereby building a psychological “firewall” against depression (Cotter & Pawelski, 2021). This aligns with the perspective of positive psychology, which states that mental health is not merely the absence of mental illness, but rather a vibrant and fulfilling life (Piepiora et al., 2025). Elderly individuals derive feelings of achievement from engaging in artistic endeavors, experience enjoyment from appreciating art, and find social support along with a sense of community through participating in collective art activities—all of which are important elements constituting subjective well-being (Cohen et al., 2007). As a person’s subjective well-being improves, so does their capability to withstand and manage adverse life events, which in turn lowers the likelihood of experiencing depression (Liang, 2024). Because both happiness and depressed or frustrated mood were assessed using single self-reported items that represent conceptually related emotional constructs, the observed indirect association through subjective well-being may partially reflect shared measurement characteristics rather than a purely substantive psychological process.

The findings did not support H3: the indirect association through the reported frequency of resting and relaxing alone was not significant. This result should not be interpreted as showing that the DRAMMA relaxation experience is irrelevant, because the CGSS item measured behavioral frequency rather than subjective psychological recovery. H4 received statistical support, but the serial indirect association through resting/relaxing frequency and subjective well-being was very small (standardized indirect effect = −0.002), indicating limited practical significance. Because receptive arts engagement is itself a form of leisure activity, the modeled arrows should not be read as a temporal sequence in which arts participation first produces a separate leisure behavior and then produces well-being. Instead, the estimates describe a model-specific pattern in which receptive arts engagement and more frequent resting/relaxing co-occurred and were associated with subjective well-being. The DRAMMA framework helps organize this interpretation, but the present cross-sectional, single-item measures do not constitute a direct test of the proposed psychological recovery experiences.

The focus on older adults also has practical significance. Later-life mental health support must address depressive symptoms alongside opportunities for well-being and social participation. Loneliness and social isolation are salient psychosocial risks in this population (Fan, 2016; Simard & Volicer, 2020; Zubatsky et al., 2020). Community-based arts opportunities may provide accessible settings for meaningful engagement and interaction. However, the CGSS exposure captured only listening to music or appreciating artworks and did not distinguish individual from group participation. Social connection was therefore not tested as a mediator. Future research should examine whether, and under what conditions, socially embedded arts engagement supports subjective well-being and is associated with fewer depressive symptoms among older adults.

At the population level, these considerations are relevant to China’s rapidly aging society. Community-based cultural services may complement existing mental health and social care by creating accessible opportunities for engagement that reflect older adults’ preferences, abilities, and local contexts. However, the present data do not identify which program features support mental health, well-being, or social connection. The findings should therefore motivate further program evaluation rather than prescribe specific interventions.

### Limitations

Despite the many advantages of this study, there are still some limitations. First, this study uses cross-sectional data. Although the mediation model describes possible statistical paths between variables, it cannot establish causal relationships. Reverse directionality is possible: older adults with fewer depressive symptoms and higher subjective well-being may be more likely to engage with the arts. Secondly, all focal variables were single-item self-reports, which may introduce social desirability, recall, and common-method biases and cannot capture the full conceptual breadth of arts engagement, relaxation, well-being, or depression. Compared with validated multi-item instruments, these single-item indicators provide limited information regarding internal consistency, measurement reliability, and construct validity. In particular, the happiness item represents only one aspect of subjective well-being, whereas the mood item reflects the self-reported frequency of depressed or frustrated mood rather than clinically assessed depression. Furthermore, because happiness and depressed or frustrated mood represent conceptually related aspects of emotional functioning and were measured simultaneously using self-report, shared conceptual variance and common-method bias may have strengthened the observed indirect association through subjective well-being. Therefore, the mediation findings should be interpreted cautiously and require confirmation using validated multidimensional psychological measures in future longitudinal studies. For example, reports of arts engagement and depressive symptom frequency may be influenced by the respondent’s current emotional state. Third, the exposure covered only listening to music or appreciating artworks. The available measure did not provide information about other art forms, active versus receptive participation, individual versus group engagement, or the depth and quality of engagement. It also did not assess structured arts programs or art therapy. Accordingly, the findings should be interpreted as applying to receptive, everyday arts engagement rather than to art activities or art therapy broadly. Finally, despite the large sample size, the presence of many missing values in key variables means that the analyzed sample may differ systematically from excluded respondents in unmeasured ways, which could affect generalizability. A further limitation is the potential for residual confounding. The models did not adjust for several plausible common causes of receptive arts engagement and mental health, including income, retirement or employment status, loneliness, social participation, chronic illness, disability, mobility limitations, cognitive functioning, living arrangement, and access to cultural institutions. These factors may influence both opportunities or motivation to engage with the arts and depressive symptoms or subjective well-being. Consequently, some of the estimated direct and indirect associations may reflect unmeasured differences between respondents rather than the specified pathways alone. Adjustment for broad sociodemographic characteristics and self-rated health is unlikely to capture these domains fully. The findings should therefore not be interpreted causally, and future longitudinal studies should use theoretically informed confounder selection, more comprehensive health and social measures, and sensitivity analyses to assess the robustness of the associations to unmeasured confounding. In addition, the depressive-symptom item did not measure the DSM-5-TR requirement of symptoms persisting for at least two weeks or being associated with clinically significant distress or impairment in occupational, social, or relational functioning. The observed associations therefore concern mood-frequency scores and should not be generalized to the prevalence, onset, remission, or treatment of clinically diagnosed major depressive disorder.

Future research should distinguish observational studies of everyday receptive arts engagement from evaluations of structured arts interventions or art therapy. Observational studies should measure artistic modality, frequency, duration, intensity, cumulative exposure, setting, and individual versus group engagement. Trials of therapeutic or program-based activities should additionally report the treatment goal, session length, session frequency, total number of sessions, facilitator qualifications, and intervention protocol. Longitudinal and experimental designs using multidimensional mental-health measures would then allow the associations observed here to be tested with clearer temporal, dose–response, and intervention-specific interpretations.

Finally, although the original CGSS was based on a national probability sample, the complete-case analytical sample should not be assumed to retain full national representativeness. The large amount of unavailable data for the leisure-related relaxation item may reflect questionnaire routing or structural non-administration rather than ordinary item nonresponse. Respondents with complete data may differ systematically from those excluded, resulting in potential selection bias. The generalizability of the findings is therefore limited to older respondents with complete observations on all variables included in the model. Future analyses should verify the module-assignment mechanism and compare included and excluded respondents using available sampling and sociodemographic variables.

## 6. Conclusions

Based on an analysis of the national CGSS 2023 sample, this study found that more frequent receptive arts engagement was associated with fewer depressive symptoms. Subjective well-being accounted for the largest indirect association among the pathways tested. Leisure-related relaxation did not show a significant indirect association when examined alone. A small serial indirect association was observed through leisure-related relaxation and subjective well-being. However, this model-specific association does not establish relaxation as the primary or causal mechanism of arts engagement or art therapy. Rather, it identifies one possible pathway that should be examined alongside broader psychological, biological, social, and behavioral processes in longitudinal and experimental research.

This study provides observational evidence that more frequent receptive arts engagement is associated with fewer depressive symptoms among older adults. Among the indirect pathways examined, subjective well-being accounted for the largest association, whereas the sequential pathway through leisure-related relaxation and subjective well-being was statistically significant but very small. Accordingly, these findings should be interpreted as supporting one possible psychological pathway rather than demonstrating a clinically meaningful or highly effective public-health intervention. Future longitudinal and intervention studies are needed to determine whether these associations translate into meaningful improvements in mental health outcomes.

## Figures and Tables

**Table 1:** Basic characteristics of the sample.

Variables	Category	n (%)
Gender	Male	1100 (47.2%)
	Female	1232 (52.8%)
Education	No formal education	415 (17.8%)
	Private tutoring/literacy class	19 (0.8%)
	Primary school	698 (29.9%)
	Junior high school	623 (26.7%)
	Vocational high school	10 (0.4%)
	General high school	333 (14.3%)
	Technical secondary school	95 (4.1%)
	Technical school	10 (0.4%)
	Junior college (adult higher education)	54 (2.3%)
	Junior college (regular higher education)	30 (1.3%)
	Bachelor’s degree (adult higher education)	13 (0.6%)
	Bachelor’s degree (regular higher education)	28 (1.2%)
	Master’s degree or above	3 (0.1%)
	Other	1 (0%)
Marital status	Never married	37 (1.6%)
	Cohabiting	66 (2.8%)
	Married, first marriage	1582 (67.8%)
	Remarried	48 (2.1%)
	Separated	17 (0.7%)
	Divorced	72 (3.1%)
	Widowed	510 (21.9%)
Place of household registration	Same township	1852 (79.4%)
	Other townships within the same county	361 (15.5%)
	Other counties/districts	119 (5.1%)
Self-rated health	Very unhealthy	246 (10.5%)
	Unhealthy	551 (23.6%)
	Fair	744 (31.9%)
	Healthy	577 (24.7%)
	Very healthy	214 (9.2%)

**Table 2:** Pearson correlation coefficients and the descriptive statistics.

Variables	M	SD	1	2	3	4
1. Art Activities	2.16	1.04	1			
2. Leisure and Relaxation	3.76	0.98	0.110 ***	1		
3. Subjective Well-being	3.91	0.89	0.175 ***	0.096 ***	1	
4. Depressive Symptom Score	2.25	1.2	−0.136 ***	−0.017	−0.302 ***	1

*** *p* < 0.001, n = 2332.

**Table 3:** Results of regression analysis.

Predictor Variable	Model 1 Depressive Symptoms			Model 2 Leisure and Relaxation			Model 3 Subjective Well-Being			Model 4 Depressive Symptoms		
	β	SE	*p*	β	SE	*p*	β	SE	*p*	β	SE	*p*
Gender	0.035	0.019	0.061	0.037	0.021	0.083	−0.026	0.02	0.209	0.031	0.018	0.093
Age	−0.048 *	0.019	0.01	0.063 **	0.021	0.003	0.122 ***	0.02	<0.001	−0.024	0.018	0.201
Education	−0.145 ***	0.019	<0.001	0.106 ***	0.022	<0.001	0.008	0.021	0.716	−0.141 ***	0.019	<0.001
Marital	0.006	0.019	0.736	0.022	0.022	0.313	−0.027	0.021	0.204	0.002	0.019	0.926
Household Registration Location	−0.044 *	0.018	0.017	0.017	0.021	0.422	0.063 **	0.02	0.002	−0.032	0.018	0.081
Physical Health Status	−0.43 ***	0.018	<0.001	−0.082 ***	0.021	<0.001	0.207 ***	0.02	<0.001	−0.392 ***	0.018	<0.001
Art Activities	−0.042 *	0.019	0.025	0.101 ***	0.021	<0.001	0.138 ***	0.021	<0.001	−0.013	0.019	0.472
Leisure and Relaxation							0.084 ***	0.02	<0.001	−0.002	0.018	0.907
Well-being										−0.193 ***	0.019	<0.001

* *p* < 0.05, ** *p* < 0.01, *** *p* < 0.001.

**Table 4:** Result of chain mediation analysis.

	Pathway	Effect Size	SE	95% CI
Total effect	Art activities → Depressive symptom score	−0.042	0.019	[−0.078, −0.005]
Direct effect	Art activities → Depressive symptom score	−0.013	0.019	[−0.050, 0.023]
Total indirect effect		−0.028	0.005	[−0.040, −0.019]
Indirect effect 1	Art activities → Leisure and relaxation → Depressive symptom score	0	0.002	[−0.004, 0.004]
Indirect effect 2	Art activities → Subjective well-being → Depressive symptom score	−0.027	0.005	[−0.037, −0.018]
Indirect effect 3	Art activities → Leisure and relaxation → Subjective well-being → Depressive symptom score	−0.002	0.001	[−0.003, −0.001]

## Data Availability

The data that support the findings of this study are available in CGSS2023 at http://cgss.ruc.edu.cn/English/Documentation/Sampling_Design.htm, accessed on 1 September 2026.

## Abbreviations

The following abbreviations are used in this manuscript:

CGSS	Chinese General Social Survey
DRAMMA	Detachment, Relaxation, Autonomy, Mastery, Meaning, Affiliation
SWB	Subjective well-being
